# Prognostic implications of abnormalities of chromosome 13 and the presence of multiple cytogenetic high-risk abnormalities in newly diagnosed multiple myeloma

**DOI:** 10.1038/bcj.2017.83

**Published:** 2017-09-01

**Authors:** M Binder, S V Rajkumar, R P Ketterling, P T Greipp, A Dispenzieri, M Q Lacy, M A Gertz, F K Buadi, S R Hayman, Y L Hwa, S R Zeldenrust, J A Lust, S J Russell, N Leung, P Kapoor, R S Go, W I Gonsalves, R A Kyle, S K Kumar

**Affiliations:** 1 Division of Hematology, Mayo Clinic, Rochester, MN, USA; 2 Division of Cytogenetics, Department of Laboratory Medicine and Pathology, Mayo Clinic, Rochester, MN, USA; 3 Division of Nephrology and Hypertension, Mayo Clinic, Rochester, MN, USA

## Abstract

Fluorescence *in situ* hybridization evaluation is essential for initial risk stratification in multiple myeloma. While the presence of specific cytogenetic high-risk abnormalities (HRA) is known to confer a poor prognosis, less is known about the cumulative effect of multiple HRA. We studied 1181 patients with newly diagnosed multiple myeloma who received novel agents as first-line therapy. High-risk abnormalities were defined as t(4;14), t(14;16), t(14;20) and del(17p). There were 884 patients (75%) without any HRA and 297 patients (25%) with HRA, including 262 (22%) with one HRA and 35 (3%) with two HRA. The presence of one HRA (versus zero, hazard ratio (HR) 1.65, 95% confidence interval (CI) 1.32–2.05, *p*<0.001) and the presence of two HRA (versus zero, HR 3.15, 95% CI 2.00–4.96, *p*<0.001) were of prognostic significance after adjusting for other prognostic factors. Abnormalities of chromosome 13 were of prognostic significance independent of the established HRA: Monosomy 13 (HR 1.27, 95% CI 1.04–1.56, *P*=0.022) and del(13q) (HR 0.48, 95% CI 0.28–0.81, *P*=0.006) with opposite effects. Patients with HRA experienced worse overall survival suggesting a cumulative adverse effect of multiple HRA. Abnormalities of chromosome 13 were of prognostic significance after adjusting for other prognostic factors.

## Introduction

Cytogenetic evaluation using fluorescence *in situ* hybridization (FISH) at the time of diagnosis is essential for initial risk stratification in multiple myeloma.^1, 2^ The presence of specific cytogenetic high-risk abnormalities (HRA) including t(4;14), t(14;16), t(14;20), del(17p) and dup(1q) is known to confer a poor prognosis.^3^ Proteasome inhibition seems to improve outcomes in patients with t(4;14) and del(17p) while the effect on other HRA is less clear.^4, 5^ These improvements in outcomes were observed for t(4;14) and del(17p) occurring in isolation but not for the presence of both abnormalities.^6^ Patients are usually considered high-risk based on the presence of any one of the known HRA, given the observed adverse impact on survival outcomes. An exact assessment of the impact of specific cytogenetic abnormalities is difficult, especially when these abnormalities are considered in isolation. Deletions involving chromosome 13 as identified by chromosome studies were one of the first recognized adverse prognostic factors in patients with newly diagnosed multiple myeloma.^7, 8, 9, 10^ It was only later that distinct patterns of co-segregation with other adverse cytogenetic abnormalities such as t(4;14) and del(17p) were observed and the potential for unmeasured confounding in previous studies became evident.^11, 12, 13, 14, 15^ The relatively small number of patients with each HRA in single-center cohorts and prospective clinical trials often precludes meaningful comparative analyses including the adjustment for potential confounding factors. Few studies specifically investigated the independent contribution of specific HRA and some studies were carried out before the routine use of immunomodulators and proteasome inhibitors.^16, 17, 18^ There were considerable differences in study populations and the assessment of HRA in terms of methodology and comprehensiveness. Attempts to adjust for potential confounding factors including co-existent HRA and other known prognostic factors was variable in these studies. Given the aforementioned circumstances, there is uncertainty about the individual contribution of specific HRA and the cumulative effect of multiple HRA at the time of diagnosis in the era of novel agents. We therefore aimed to evaluate the prognostic implications of the presence of chromosome 13 abnormalities and the established HRA in patients with newly diagnosed multiple myeloma treated with novel agents, taking into account other known prognostic factors.

## Patients and methods

We retrospectively studied 1181 patients who were diagnosed with multiple myeloma between July 2005 and July 2015 at Mayo Clinic Rochester, underwent FISH evaluation within six months of diagnosis, and received first-line therapy with at least one novel agent (immunomodulator or proteasome inhibitor).

HRA were defined as t(4;14), t(14;16), t(14;20) and del(17p). Only patients diagnosed after August 2014 (12% of the cohort) routinely underwent evaluation for dup(1q); therefore, data on dup(1q) were not included in the analysis. Bone marrow aspirates were evaluated by immunofluorescent-labeled antibodies against cytoplasmic kappa and lambda immunoglobulin light chains to selectively identify the plasma cell population. The following FISH probes were evaluated, including: translocations of the immunoglobulin heavy chain gene region (IGH) using a break-apart IGH probe and a dual-fusion FISH (D-FISH) probe for the five common IGH partners (CCND1, CCND3, MAF, MAFB, FGFR3); Centromere probes (D3Z1, D7Z1, D9Z1, D15Z4, D17Z1) for copy number gain of chromosomes 3, 7, 9, 15 and 17; Locus-specific probe strategies for 17p deletion (D17Z1, TP53), 1q duplication (TP73, CKS1B), and monosomy 13/deletion 13q (RB1, LAMP1) with del(13q) referring to the loss of RB1. For each probe set, 50–100 plasma cells were evaluated if available, with a minimum of 25 plasma cells generally evaluated per FISH hybridization site. For D-FISH probes, at least three abnormal cells had to be identified to be considered positive. For trisomies, at least five cells with trisomy had to be identified to be considered positive. For monosomies and deletions, at least five cells with monosomy and at least seven cells with deletion had to be identified to be considered positive. Less than 1% plasma cells in the bone marrow were considered insufficient for FISH testing. The FISH probes used for each sample included chromosome 13 (RB1/LAMP1; Abbott Molecular, Des Plaines, IL, USA), chromosome 17 (TP53/D17Z1; Abbott Molecular), chromosomes 3/7 (D3Z1/D7Z1; Abbott Molecular), chromosomes 9/15 (D9Z1/D15Z4; Abbott Molecular), MYC (5'/3' MYC break-apart; Abbott Molecular), IGH (3'/5' IGH break-apart; Homebrew), t(4;14) (FGFR3/IGH dual fusion; Abbott Molecular), t(6;14) (CCND3/IGH dual fusion; Homebrew), t(11;14) (CCND1/IGH dual fusion; Abbott Molecular), t(14;16) (IGH/MAF dual fusion; Abbott Molecular) and t(14;20) (IGH/MAFB dual fusion; Homebrew). For translocation (dual fusion) probe sets, at least three abnormal cells had to be present to be considered positive. For trisomies at least five cells needed to contain the trisomy to be considered positive. For monosomies, the number of affected cells required for positivity was at least five cells for chromosomes 13 and 17, and at least seven cells for all other chromosomes.

Kaplan–Meier overall and progression-free survival estimates were calculated and the log-rank test was used to compare overall and progression-free survival in patients with and without HRA (stratified by the number of HRA).^19^ Multivariable-adjusted Cox regression models were used to assess the effect of HRA on overall and progression-free survival adjusting for age, sex, International Staging System (ISS)^20^ or revised ISS (R-ISS)^21^ stage (categorical variable with reference category ISS I and R-ISS I, respectively), and first-line therapy (immunomodulator, proteasome inhibitor, upfront autologous hematopoietic stem cell transplantation).^22^ Fisher’s exact test was used to compare proportions in subgroups. All hypothesis tests were two-sided, *P*-values below 0.05 were considered statistically significant. The Stata software (version 13.1, StataCorp, College Station, TX, USA) was used for statistical analysis.

## Results

There were 1181 patients with complete clinical, cytogenetic (FISH), and overall survival data available. The median age at diagnosis was 65 years (28–95), 708 (60%) of the patients were male. The most common first-line therapies were lenalidomide+dexamethasone (*n*=471), cyclophosphamide+bortezomib+dexamethasone (*n*=252), bortezomib+lenalidomide+dexamethasone (*n*=143) and bortezomib+dexamethasone (*n*=112). Sixty-six patients received thalidomide as part of their first-line treatment.

There were 332 HRA in 297 patients (25% of the entire cohort): 170 (51%) del(17p), 110 (33%) t(4;14), 44 (13%) t(14;16) and 8 (3%) t(14;20). Of the 262 patients with one HRA: 135 (52%) had del(17p) and 127 (48%) had a high-risk translocation (HRT). Thirty-five patients had both del(17p) and an HRT (two HRA). All patients received at least one novel agent as part of their first-line treatment: 590 (50%) received a proteasome inhibitor, 416 (35%) received an immunomodulator and 175 (15%) received both. Four hundred and fifty of the patients (38%) underwent autologous hematopoietic stem cell transplantation as part of their first-line treatment. The median overall survival for the entire cohort (*n*=1181) was 6.6 years (6.0–8.0). There was an association between higher ISS stages and higher numbers of HRA in the entire cohort (*n*=1181, *P*=0.004). Demographic, clinical and cytogenetic characteristics of the entire cohort stratified by the number of HRA are summarized in Table 1.

### Contribution of the number of HRA

**Figure 1:**
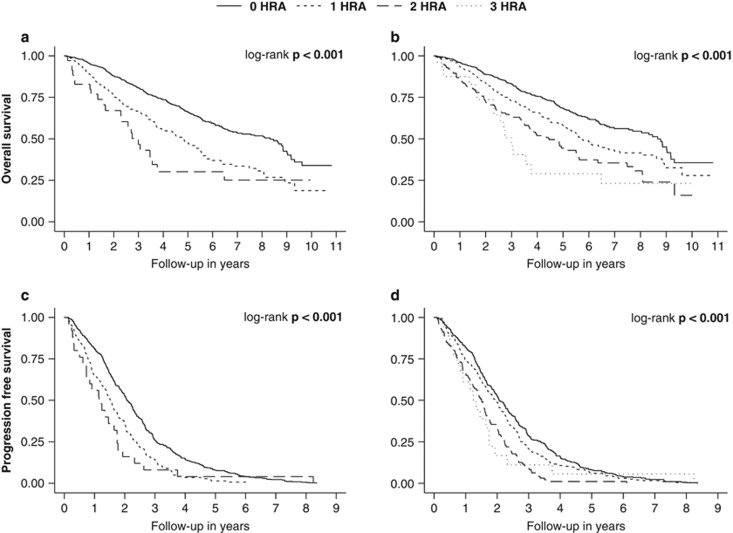
Kaplan–Meier overall (*n*=1181) and progression-free survival (*n*=660) estimates for patients with newly diagnosed multiple myeloma stratified by the number of cytogenetic high-risk abnormalities (HRA): (**a**,**c**) established HRA only, (**b**,**d**) considering monosomy 13 as an additional HRA.

Overall survival was significantly shorter in patients with one HRA (compared to zero, median 4.9 versus 8.3 years, *P*<0.001) and in patients with two HRA (compared to zero, median 2.7 versus 8.3 years, *P*<0.001). Progression-free survival was significantly shorter in patients with one HRA (compared to zero, median 1.5 versus 2.1 years, *P*<0.001) and in patients with two HRA (compared to zero, median 1.2 versus 2.1 years, *P*=0.007). Figure 1 shows the Kaplan–Meier overall and progression-free survival estimates stratified by the number of HRA in the entire cohort (*n*=1181 and *n*=660; please see Supplementary Figure 1 for an extended patient cohort). Both the presence of one HRA (compared to zero) and the presence of two HRA (compared to zero) were of prognostic significance after adjusting for age, sex, ISS stage and first-line therapy (Table 2). When adjusting for the revised ISS instead of the ISS the hazard was slightly attenuated for the same analysis (Table 2). Effect estimates for the ISS and R-ISS as well as other statistically significant prognostic factors are shown in Supplementary Table 1.

The effect of upfront autologous hematopoietic stem cell transplantation was not significantly different across the strata (*P*-value for interaction 0.159). When stratifying on the number of HRA there was a trend towards a more profound effect of upfront transplantation in those with higher numbers of HRA: Zero HRA (hazard ratio (HR) 0.53, 95% confidence interval (CI) 0.40–0.70, *P*<0.001, *n*=884), one HRA (HR 0.40, 95% CI 0.24–0.66, *P*<0.001, *n*=262) and two HRA (HR 0.27, 95% CI 0.09–0.89, *P*=0.031, *n*=35) after adjusting for age, sex, ISS stage and first-line therapy. Because of the small sample size in the group of two HRA (*n*=35) sensitivity analyses were carried out: The estimates remained stable when only adjusting for IIS stage (HR 0.21, 95% CI 0.07–0.66, *P*=0.008, *n*=35), only adjusting for age, sex and ISS stage (HR 0.24, 95% CI 0.07–0.76, *P*=0.015, *n*=35) and when only adjusting for ISS stage and first-line treatment (HR 0.20, 95% CI 0.06–0.62, *P*=0.006, *n*=35). Different first-line regimens were not associated with overall but with progression-free survival; multivariable-adjusted estimates for the effect of the different first-line regimens (immunomodulator, proteasome inhibitor, both) are shown in Supplementary Table 2.

### Contribution of specific HRA

The presence of del(17p) and HRT was independently associated with increased hazard after adjusting for age, sex, ISS stage and first-line therapy (Table 2). When adjusting for the revised ISS instead of the ISS the hazard was slightly attenuated for the same analysis (Table 2). The magnitude of these adverse effects was similar for del(17p) and HRT. The hazard for patients with an isolated HRT (compared to an isolated del(17p), HR 1.08, 95% CI 0.75–1.56, *P*=0.681, *n*=262) was similar after adjusting for age, sex, ISS stage and first-line therapy. The hazard was significantly increased for patients with the combination of del(17p) and an HRT compared to both an isolated del(17p) and an isolated HRT after adjusting for age, sex, ISS stage and first-line therapy (Table 2).

### Abnormalities of chromosome 13

**Figure 2:**
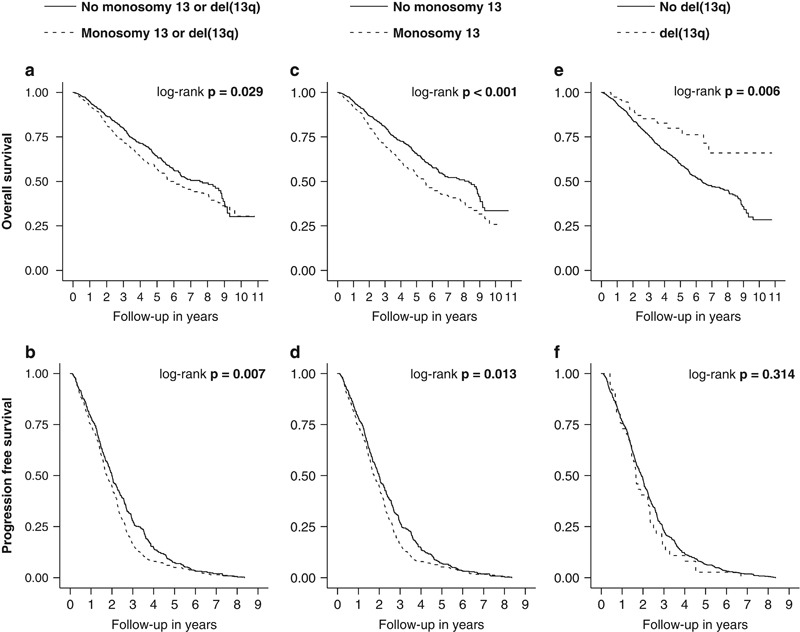
Kaplan–Meier overall (*n*=1181) and progression-free survival (*n*=660) estimates for patients with newly diagnosed multiple myeloma stratified by the presence of (**a**,**b**) monosomy 13 or del(13q), (**c**,**d**) monosomy 13, and (**e**,**f**) del(13q).

Four hundred and eleven (35%) of the patients had monosomy 13, 73 (6%) had del(13q) and 9 (1%) had both. The distribution of the established HRA was similar among patients with monosomy 13 and del(13q) (Supplementary Table 3). The presence (compared to the absence) of monosomy 13 was associated with significantly shorter overall (median 5.5 versus 8.3 years, *P*<0.001) and progression-free survival (median 1.75 versus 2.00 years, *P*=0.013) while the presence (compared to the absence) of del(13q) was associated with significantly longer overall (median not reached versus 6.4 years, *P*=0.006) but not progression-free survival (median 1.93 versus 1.66 years, *P*=0.314). Figure 2 shows the Kaplan–Meier overall and progression-free survival estimates stratified by the presence of monosomy 13 and del(13q) in the entire cohort (*n*=1181 and *n*=660).

In regards to overall survival, the hazard for patients with either monosomy 13 or del(13q) (compared to neither abnormality, HR 1.09, 95% CI 0.88–1.34, *P*=0.425, *n*=1181) was similar after adjusting for age, sex, ISS stage, first-line therapy and the presence of HRA. Both the presence of monosomy 13 (compared to the absence, HR 1.27, 95% CI 1.04–1.56, *P*=0.022, *n*=1181) and the presence of del(13q) (compared to the absence, HR 0.48, 95% CI 0.28–0.81, *P*=0.006, *n*=1181) were of prognostic significance after adjusting for age, sex, ISS stage, first-line therapy and the presence of the established HRA. This protective effect of del(13q) remained similar (HR 0.48, 95% CI 0.28–0.80, *P*=0.005) when additionally adjusting for the presence of t(11;14) and t(6;14). When adjusting for the revised ISS instead of the ISS the hazard was similar for the same analyses: Presence of monosomy 13 (compared to the absence, HR 1.26, 95% CI 1.02–1.56, *P*=0.032, *n*=1087) and presence of del(13q) (compared to the absence, HR 0.45, 95% CI 0.26–0.79, *P*=0.005, *n*=1087).

In regards to progression-free survival, the hazard for patients with either monosomy 13 or del(13q) (compared to neither abnormality, HR 1.16, 95% CI 0.99–1.38, *P*=0.073, *n*=660) was similar after adjusting for age, sex, ISS stage, first-line therapy and the presence of HRA. Neither the presence of monosomy 13 (compared to the absence, HR 1.17, 95% CI 0.99–1.38, *P*=0.061, *n*=660) nor the presence of del(13q) (compared to the absence, HR 1.04, 95% CI 0.74–1.47, *P*=0.807, *n*=660) were of prognostic significance after adjusting for age, sex, ISS stage, first-line therapy and the presence of the established HRA. Figure 1 shows the Kaplan–Meier overall and progression-free survival estimates stratified by the number of HRA in the entire cohort (*n*=1181 and *n*=660; please see Supplementary Figure 1 for an extended patient cohort) when considering monosomy 13 as an additional HRA.

## Discussion

The introduction of novel agents including immunomodulators and proteasome inhibitors has led to significant improvements in overall survival for patients with multiple myeloma. Despite these advances, patients with high-risk multiple myeloma do not seem to benefit from these new treatments as much as patients with standard-risk disease.^23^ There is evidence that proteasome inhibition is beneficial both for patients with an isolated t(4;14) and an isolated del(17p) but not for patients with both cytogenetic HRA.^4, 5, 6^ The relatively small number of patients with each HRA in individual prospective clinical trials has limited the ability to carry out meaningful comparative analyses. In a retrospective analysis of 242 patients with either t(4;14) or del(17p), 25 patients (10%) presented with both abnormalities.^17^ Their analysis focused on the effects of further abnormalities in addition to an isolated t(4;14) or isolated del(17p) demonstrating specific deletions, numerical chromosomal abnormalities and patterns of co-segregation modulating survival outcomes in patients with high-risk multiple myeloma. The focus on these two well-established cytogenetic HRA likely explains the lower prevalence of multiple HRA compared to our cohort that also included patients with t(14;16) and t(14;20). An ameliorating effect of concomitant trisomies in patients with newly diagnosed high-risk multiple myeloma was seen in one study^24^ but not in others.^17, 25^

In this study we examined the effect of multiple HRA at the time of diagnosis on overall and progression-free survival. We observed an association between higher numbers of HRA and higher ISS stages, suggesting that the more aggressive disease biology was reflected by higher tumor burden and more extensive end-organ damage in this cohort. The co-segregation of these adverse prognostic factors emphasizes the need to adjust for these potential confounding factors when attempting to estimate the contribution of specific HRA either in isolation or in combination. While both isolated HRT and isolated del(17p) seemed to have a similar impact on overall survival, concurrent, multiple HRA further negatively affected overall survival. This finding is consistent with a study examining the effect of multiple HRA in patients treated with non-contemporary chemotherapy regimens in the first-line setting with or without subsequent autologous hematopoietic stem cell transplantation.^16^ In the aforementioned study the increase in hazard associated with more than one HRA (compared to one HRA) was approximately 2.5-fold, which is slightly lower than our estimate. In our cohort approximately one in 33 patients presented with two HRA at the time of diagnosis and their hazard of subsequent mortality was approximately two-fold compared to a single HRA and three-fold compared to the absence of HRA when adjusting for potential confounding factors. Upfront autologous hematopoietic stem cell transplantation remains the preferred treatment strategy in eligible patients with newly diagnosed multiple myeloma across age groups.^26, 27, 28^ In this study, upfront transplantation was associated with improved overall survival regardless of the number of HRA. However there was a trend towards greater benefit in those with a higher number of HRA, suggesting the ability to overcome some of the high-risk disease features with more intensive therapy. This may in part be related to deeper and faster responses to treatment but at the same time there is the potential for residual confounding in this small and highly selected patient group.

The combination of del(17p) and a HRT (compared to either isolated HRA) was associated with an approximately two-fold increase in hazard, again suggesting similar additive adverse effects of these two most common HRA. The calculated hazard ratios represent conservative effect estimates and likely slightly underestimate the true effect of multiple HRA since we did not include data on dup(1q) in this analysis. The point estimates for del(17p) and HRT indicated an adverse effect of similar magnitude when adjusting for potential confounding factors. This is consistent with a study reporting unadjusted estimates of similar magnitude for del(17p), t(4;14) and dup(1q) concluding that these HRA are of similar prognostic significance.^18^ Our analysis did not allow for a complete assessment of all potential HRA including the more recently described dup(1q) and del(1p32).^29^ Future studies will need to take into account these emerging factors in order to fully understand the individual contributions of each HRA.

The monosomy of chromosome 13 and deletions of its long arm had been regarded adverse prognostic factors in numerous studies when defined by karyotype^7, 8, 9, 10^ before the potential confounding effect of other HRA such as t(4;14) and del(17p) became apparent.^11, 12, 13, 14, 15^ In this study we have a novel observation of differential effects of monosomy 13 (adverse) and partial deletion (protective) of chromosome 13q on overall survival. These effects were independent of known prognostic factors including the established HRA. The aforementioned differential effects on overall survival were not evident when grouping the partial deletion of 13q and monosomy 13 together (as frequently done in previous studies), which may explain why this contrasting effect has not been observed before. Despite a total number of 1181 patients, the subgroup of patients with del(13q) was small (*n*=82, 7%) and therefore studies with smaller sample sizes may not have had the power to detect the impact on overall survival. This may also explain the lack of statistical significance in some of our progression-free survival analyses, given the lower sample size for that endpoint. Moreover, most of the literature on monosomy 13 and del(13q) predates the introduction of the novel agents and abnormalities of chromosome 13 may not have been as relevant in patients treated with those regimens. The adverse effect of monosomy 13 on overall survival was independent and approximately half the magnitude of the established HRA. When considering monosomy 13 as an additional HRA it was helpful in further sub-stratifying patients who were considered intermediate based on their number of HRA before. Several explanations for the observed negative effects of chromosome 13 abnormalities were proposed in the past, including reduced expression of *RB1*, higher disease burden and higher proliferation rate.^10, 30^ It is conceivable that a higher proliferation rate translates into better response to treatment in the era of highly active first-line therapies (that is, the combination of novel agents), which is known to confer a better prognosis.^31^ In chronic lymphocytic leukemia del(13q) represents the most common detected abnormality and, in isolation, is associated with a better prognosis.^32^ Most studies did not analyze del(13q) separately from monosomy 13 and given the loss of the whole chromosome in the latter the identification of one or more causative genes poses a challenge. The observed differential effect of monosomy 13 and partial deletions of chromosome 13q will need to be validated in other studies to confirm its prognostic significance and the underlying mechanism remains to be elucidated.

In this study, each additional HRA had an independent adverse effect and patients with two HRA at the time of diagnosis experienced a median overall survival of approximately 3 years. The existence of this ultra-high-risk population defined by two HRA raises the question whether or not there is a ‘double hit’ biology at work as recognized in other lymphoid malignancies.^33^ Our findings support the notion that there has been an improvement in overall survival since the introduction of novel agents even among patients with high-risk disease; however, the relative impact of HRA remains unchanged. Approximately one in four patients with newly diagnosed multiple myeloma presented with one HRA, approximately one in 33 with two. The latter patients experienced worse overall survival suggesting a cumulative adverse effect of multiple HRA in the era of novel agents. We observed a differential effect of partial 13q deletions and monosomy 13 on overall survival, which was less pronounced but independent of the established prognostic factors.

## Figures and Tables

**Table 1:** Demographic, clinical and cytogenetic characteristics of 1181 patients with newly diagnosed multiple myeloma stratified by the number of cytogenetic high-risk abnormalities (HRA)

	*0 HRA* n=*884*	*1 HRA* n=*262*	*2 HRA* n=*35*
Men (*n* (%))	540 (61)	147 (56)	21 (60)
Age at diagnosis (years)	65 (28–95)	65 (32–91)	63 (48–80)
Follow-up (years)	3.3 (0.1–10.8)	2.5 (0.2–10.7)	2.6 (0.1–10.0)
Overall survival (years)	8.3 (6.7–8.9)	4.9 (3.7–5.5)	3.0 (1.7–3.8)
			
*ISS stage at diagnosis (n (%))*			
I	270 (31)	66 (25)	5 (14)
II	354 (40)	99 (38)	11 (32)
III	260 (29)	97 (37)	19 (54)
			
*Overall survival by ISS stage (years (95% CI))*			
I	9.2 (8.6–NR)	NR (3.5–NR)	6.5 (1.1–NR)
II	8.5 (6.6–8.9)	4.9 (3.7–5.8)	3.5 (1.0–NR)
III	5.0 (4.2–5.6)	3.7 (2.1–5.3)	2.6 (0.4–3.6)
			
*Cytogenetic high-risk abnormalities (n (%))*			
del(17p)	0 (0)	135 (52)	35 (100)
High-risk translocation	0 (0)	127 (48)	35 (100)
t(4;14)	0 (0)	87 (33)	23 (66)
t(14;16)	0 (0)	32 (12)	12 (34)
t(14;20)	0 (0)	8 (3)	0 (0)
			
*First-line treatment (n (%))*			
Immunomodulator	483 (55)	96 (37)	11 (31)
Proteasome inhibitor	294 (33)	111 (42)	11 (31)
Both	107 (12)	55 (21)	13 (38)
Upfront ASCT	348 (40)	91 (35)	11 (31)

Abbreviations: ASCT, Autologous hematopoietic stem cell transplantation; ISS, International Staging System; NR, not reached. Data are given as median (range) unless denoted otherwise.

**Table 2:** Effect estimates from multivariable-adjusted Cox regression models for the effect of cytogenetic high-risk abnormalities on overall survival in the entire cohort and patient subgroups

*Parameter*	*Reference*	*HR (95% CI)*	P*-value*
*Effect of multiple HRA in the entire cohort (using ISS, n=1181)*			
1 HRA	0 HRA	1.65 (1.32–2.05)	<0.001
2 HRA	0 HRA	3.15 (2.00–4.96)	<0.001
			
*Effect of multiple HRA in the entire cohort (using R-ISS, n=1087)*			
1 HRA	0 HRA	1.47 (1.16–1.86)	0.001
2 HRA	0 HRA	2.69 (1.69–4.30)	<0.001
			
*Effect of specific HRA in the entire cohort (using ISS, n=1181)*			
del(17p)	Absence of del(17p)	1.64 (1.29–2.08)	<0.001
HRT	Absence of HRT	1.78 (1.39–2.30)	<0.001
			
*Effect of specific HRA in the entire cohort (using R-ISS, n=1087)*			
del(17p)	Absence of del(17p)	1.49 (1.16–1.91)	0.002
HRT	Absence of HRT	1.62 (1.24–2.11)	<0.001
			
*Effect of specific additional HRA (using ISS, n=164 and n=154)*			
del(17p)+HRT	Isolated del(17p)	2.08 (1.19–3.63)	0.010
del(17p)+HRT	Isolated HRT	1.86 (1.07–3.22)	0.027

Abbreviations: CI, confidence interval; HR, hazard ratio; HRA, cytogenetic high-risk abnormality. HRT: High-risk translocation.

All models were adjusted for age, sex, International Staging System (ISS) or revised ISS (R-ISS) stage, and first-line therapy (immunomodulator, proteasome inhibitor, upfront autologous hematopoietic stem cell transplantation).
