# Supplementary material for: Prognostic implications of abnormalities of chromosome 13 and the presence of multiple cytogenetic high-risk abnormalities in newly diagnosed multiple myeloma
Source: Blood Cancer J. 2017 Sep 1;7(9):e600–. doi: 10.1038/bcj.2017.83 (PMC5709752; doi:10.1038/bcj.2017.83)
Supplement: Supplementary Figure 1 Legend [file bcj201783x1.docx]

**Supplemental Figure 1** Kaplan-Meier overall survival estimates for 1553 patients (the 1181 included patients plus 372 additional patients with missing data on other prognostic factors) with newly diagnosed multiple myeloma stratified by the number of cytogenetic high-risk abnormalities (HRA): **[A]** and **[C]** established HRA only, **[B]** and **[D]** considering monosomy 13 as an additional HRA.
