# Supplementary figures and images for: Prognostic implications of abnormalities of chromosome 13 and the presence of multiple cytogenetic high-risk abnormalities in newly diagnosed multiple myeloma
Source: Blood Cancer J. 2017 Sep 1;7(9):e600–. doi: 10.1038/bcj.2017.83 (PMC5709752; doi:10.1038/bcj.2017.83)

— 0 HRA    - - - 1 HRA    - - - 2 HRA    ..... 3 HRA

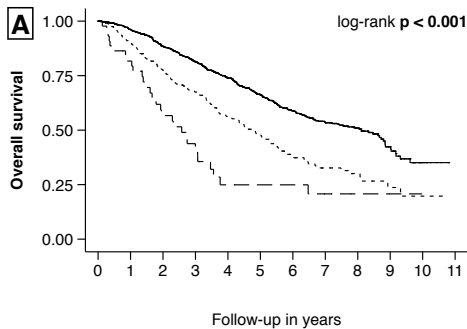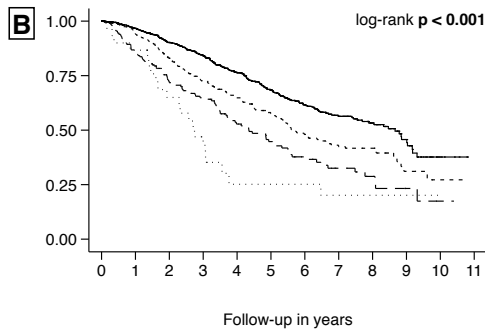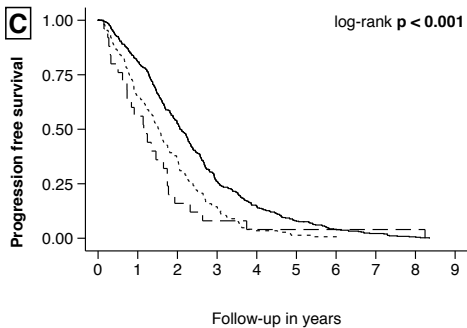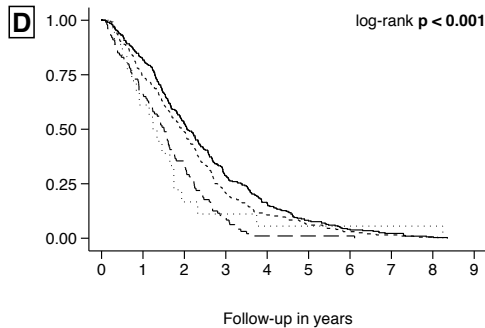

Supplement: Supplementary Figure 1 [file bcj201783x2.pdf]
