# Supplementary material for: Prognostic implications of abnormalities of chromosome 13 and the presence of multiple cytogenetic high-risk abnormalities in newly diagnosed multiple myeloma
Source: Blood Cancer J. 2017 Sep 1;7(9):e600–. doi: 10.1038/bcj.2017.83 (PMC5709752; doi:10.1038/bcj.2017.83)
Supplement: Supplementary Table 1 [file bcj201783x3.docx]

| **Supplemental Table 1** Effect estimates from multivariable-adjusted Cox regression models for the effect of the number of cytogenetic high-risk abnormalities on overall survival in the entire cohort including all statistically significant factors. | | | | | |
| --- | --- | --- | --- | --- | --- |
|  |  |  |  |  |  |
| **Parameter** |  | **Reference** | **HR (95% CI)** | **p-value** |  |
|  |  |  |  |  |  |
| *Effect of multiple HRA in the entire cohort (using ISS, n=1181)* | | | | | |
|  |  |  |  |  |  |
| **1 HRA** |  | 0 HRA | 1.65 (1.32-2.05) | <0.001 |  |
| **2 HRA** |  | 0 HRA | 3.15 (2.00-4.96) | <0.001 |  |
|  |  |  |  |  |  |
| ISS II |  | ISS I | 1.39 (1.06-1.82) | 0.018 |  |
| ISS III |  | ISS I | 2.29 (1.74-3.00) | <0.001 |  |
|  |  |  |  |  |  |
| Age at diagnosis |  | 1-year increase | 1.03 (1.02-1.04) | <0.001 |  |
| Immunomodulator |  | No immunomodulator | 0.67 (0.46-0.96) | 0.028 |  |
| Upfront ASCT |  | No upfront ASCT | 0.48 (0.38-0.61) | <0.001 |  |
|  |  |  |  |  |  |
| *Effect of multiple HRA in the entire cohort (using R-ISS, n=1087)* | | | | |  |
|  |  |  |  |  |  |
| **1 HRA** |  | 0 HRA | 1.47 (1.16-1.86) | 0.001 |  |
| **2 HRA** |  | 0 HRA | 2.69 (1.69-4.30) | <0.001 |  |
|  |  |  |  |  |  |
| ISS II |  | ISS I | 1.34 (0.99-1.80) | 0.057 |  |
| ISS III |  | ISS I | 2.02 (1.42-2.89) | <0.001 |  |
|  |  |  |  |  |  |
| Age at diagnosis |  | 1-year increase | 1.03 (1.02-1.05) | <0.001 |  |
| Immunomodulator |  | No immunomodulator | 0.71 (0.49-1.03) | 0.069 |  |
| Upfront ASCT |  | No upfront ASCT | 0.51 (0.40-0.65) | <0.001 |  |
|  |  |  |  |  |  |
| All models were adjusted for age, sex, International Staging System (ISS) or revised ISS (R-ISS) stage, and first-line therapy (immunomodulator, proteasome inhibitor, upfront autologous hematopoietic stem cell transplantation). HRA: Cytogenetic high-risk abnormality. HRT: High-risk translocation. | | | | | |
