# Supplementary material for: Prognostic implications of abnormalities of chromosome 13 and the presence of multiple cytogenetic high-risk abnormalities in newly diagnosed multiple myeloma
Source: Blood Cancer J. 2017 Sep 1;7(9):e600–. doi: 10.1038/bcj.2017.83 (PMC5709752; doi:10.1038/bcj.2017.83)
Supplement: Supplementary Table 2 [file bcj201783x4.docx]

| **Supplemental Table 2** Effect estimates from multivariable-adjusted Cox regression models for the effect of different first-line therapies. | | | | | |
| --- | --- | --- | --- | --- | --- |
|  |  |  |  |  |  |
| **Parameter** |  | **Reference** | **HR (95% CI)** | **p-value** |  |
|  |  |  |  |  |  |
| *Overall survival (n=1181)* | | | | | |
|  |  |  |  |  |  |
| **Proteasome inhibitor only** |  | Immunomodulator only | 1.15 (0.91-1.44) | 0.233 |  |
| **Both** |  | Immunomodulator only | 0.78 (0.56-1.09) | 0.145 |  |
|  |  |  |  |  |  |
| *Progression free survival (n=660)* | | | | |  |
|  |  |  |  |  |  |
| **Proteasome inhibitor only** |  | Immunomodulator only | 1.83 (1.51-2.20) | <0.001 |  |
| **Both** |  | Immunomodulator only | 1.54 (1.20-1.98) | 0.001 |  |
|  |  |  |  |  |  |
| All models were adjusted for age, sex, International Staging System (ISS) stage, upfront autologous hematopoietic stem cell transplantation, and the presence of cytogenetic high-risk abnormalities. | | | | | |
