# Supplementary material for: Prognostic implications of abnormalities of chromosome 13 and the presence of multiple cytogenetic high-risk abnormalities in newly diagnosed multiple myeloma
Source: Blood Cancer J. 2017 Sep 1;7(9):e600–. doi: 10.1038/bcj.2017.83 (PMC5709752; doi:10.1038/bcj.2017.83)
Supplement: Supplementary Table 3 [file bcj201783x5.docx]

| **Supplemental Table 3** Distribution of cytogenetic high-risk abnormalities among patients with chromosome 13 abnormalities stratified by the type of abnormality. | | | | |
| --- | --- | --- | --- | --- |
|  | | | | |
| **HRA** | **Monosomy 13** | **del(13q)** | **p-value** |  |
|  |  |  |  |  |
| **del(17p)** | 81/411 (20%) | 14/73 (19%) | 1.000 |  |
| **HRT** | 110/411 (27%) | 13/73 (18%) | 0.111 |  |
| t(4;14) | 76/411 (19%) | 7/73 (10%) | 0.065 |  |
| t(14;16) | 27/411 (7%) | 6/73 (8%) | 0.614 |  |
| t(14;20) | 7/411 (2%) | 0/73 (0%) | 0.601 |  |
|  | | | | |
| Data are given as proportion (percent) unless denoted otherwise. | | | | |
